# Anti-interleukin-6 therapy for treatment of high platelet counts in cGMP-dependent protein kinase I gene-targeted mice

**DOI:** 10.1186/2050-6511-14-S1-P80

**Published:** 2013-08-29

**Authors:** Lin Zhang, Robert Lukowski, Florian Gaertner, Michael Lorenz, Kyle R Legate, Katrin Domes, Elisabeth Angermeier, Franz Hofmann, Steffen Massberg

**Affiliations:** 1 Medizinische Klinik und Poliklinik I, Klinikum der Universität, Ludwig-Maximilians-Universität, München, Germany; 2 Forschergruppe 923, Institut für Pharmakologie und Toxikologie, Technische Universität München, München, Germany; 3 Pharmakologie, Toxikologie und Klinische Pharmazie, Institut für Pharmazie, Universität Tübingen, Tübingen, Germany; 4 Center for NanoScience, Department of Applied Physics, Ludwig-Maximilians-Universität, München, München, Germany

## Background

The cyclic guanosine-3',5'-monophosphate (cGMP)/cGMP-dependent protein kinase type I (cGKI) pathway is a potent negative regulator of platelet adhesion and aggregation [1]; however, the role of cGMP/cGKI for platelet biogenesis *in vivo* is unclear.

## Results

Here we report thrombocytosis in conventional cGKI null mutants (cGKI^L1/L1^) and gene-targeted cGKIα/β rescue mice (referred to as cGKI-SM) with cGKI expression specifically restored in smooth muscle (SM), but not in other cell types [2-4]. In contrast, conditional knockouts lacking the cGKI protein specifically in the megakaryocyte (MK)/platelet lineage (Pf4-Cre^tg/+^; cGKI^L2/L2^) did not display a related thrombocytosis phenotype, indicating that the high platelet count of cGKI^L1/L1^ and cGKI-SM mutants is rather a reactive response than an intrinsic defect in megakaryopoiesis. In line with these findings, wild-type (WT) mice engrafted with cGKI-deficient bone-marrow (BM) cells showed full reconstitution of haematopoiesis and normal platelet counts upon myeloablative radiotherapy. Stimulation of BM-derived WT MKs using serum preparations from cGKI-SM mutants strongly accelerated megakaryopoiesis, suggesting that their high platelet counts develop in response to soluble factors. Indeed, we confirm elevated Interleukin-6 (IL-6) serum levels [5,6], a known cause for reactive thrombocytosis, in cGKI-SM mutants, whereas IL-6 was unaltered in Pf4-Cre^tg/+^; cGKI^L2/L2^ mice and cGKI-deficient BM chimaeras. Vice versa, antibody-mediated blockage of IL-6 reduced platelet counts in cGKI-SM mice, but not in WT mice.

## Conclusion

We conclude that abnormal signalling of cGMP/cGKI in non-hematopoietic cells affects thrombopoiesis via IL-6 resulting in a reactive thrombocytosis *in vivo*.
